# Doping offences in male professional tennis: how does sanction affect players’ career?

**DOI:** 10.1186/s40064-016-2765-5

**Published:** 2016-07-12

**Authors:** Javier Maquirriain, Roberto Baglione

**Affiliations:** High Performance National Sports Center, CeNARD, Buenos Aires, Argentina; Argentine Tennis Association, Chubut 415, Mayling Club de Campo Pilar 1631, Buenos Aires, Argentina; CONICET, Buenos Aires, Argentina

**Keywords:** Sport, Performance, Drug, Ranking

## Abstract

**Background:**

The use of performance enhancing drugs is still a major problem in competitive sports. Even though tennis is not affected by systematic doping, there is lack of scientific evidence on the effect of sanctions on players` career. The aim of this study was to analyze male tennis players’ career after a doping sanction.

**Methods:**

All doping offences committed by male professional tennis players during 2003–2014 were registered from the International Tennis Federation records and analyzed considering three ranking positions: at sanction date, the peak career position, and the highest position after doping violation.

**Results:**

Forty-six players (aged 26.04 ± 3.48 years) committed 47 doping offences in a 14-year period with an average ineligibility time of 11.13 ± 9.90 months. Ranking position at sanction date averaged 409.53 ± 437.53 (median 266); the highest career position averaged 201.12 ± 293.96 (median 83) and the highest career position after doping violation was 350.85 ± 441.38 (median 156). Elite players committed less doping offences than players beyond the 100° ranking position (29.78 and 70.21 %, respectively; p < 0.01). Most players (72.34 %) reached their career highest position before the doping sanction (*p* = 0.01). The average time to retirement was 35.76 ± 31.03 months, while 12 % did not return to professional tennis competition after the doping offence.

**Conclusions:**

Most suspended tennis players were not elite players. Doping sanction seems to significantly affect male tennis players’ career. Only a minority of sanctioned players could reach their peak ranking position after the doping offence and suspension may accelerate the retirement process.

## Background

The use of performance enhancing drugs is probably the major problem facing sport today. Despite intense efforts by sports organizations and the medical community to attenuate the problem, drug consumption to enhance sports performance remains widespread.

Doping issues were rarely studied in tennis. However, there is scientific evidence that tennis is a sport without systematic doping practice (Maquirriain 2010). Maquirriain (2010) studied all doping offences committed in the professional tennis circuit during 7 years. The overall incidence of positive doping samples was 0.38 % and the annual rate was 7.4 cases. Male players showed higher incidence than females and wheelchair players were at higher risk to commit a doping offence. Kondric et al. (2013) also found a low tendency regarding future doping usage among international high-level players of both sexes, although most of them are convinced that doping does exist in tennis.

Health consequences of abuse of banned substances in sport are well known. Adverse effects of anabolic steroids, stimulants, erythropoietin and other drugs have been widely reported in the sports medicine literature. Social consequences of drug abuse by athletes are less documented. Some effects of receiving a doping sanction may include: loss of sponsorship deals and income, wiping out of previous achievements, damaged relationships with friends and family, isolation from peers and sport, damage to future career prospects, and effects on emotional and psychological well-being, among others (Georgiadis and Papazoglou 2014; UK Anti-doping 2016). Mazanov (2012) has proposed that the final area of research to understand the imposition of universal sanctions would be to investigate the career of athletes who have been affected by doping rules. It has been argued that imposing the same sanction on two different athletes could virtually end the career of one while amounting to no more than an extended vacation in the career of other (Houlihan 2003). Question such as whether they returned to competition, how long they continued to compete, how the sanction affected their financially, and whether they were able to return to the same level as the previously had achieved would all give some indictor of the effect of the sanction (Mazanov 2012).

Finally, to the best of our knowledge, the effect of doping on tennis players’ performance has not been yet investigated. The objective of this study was to analyze male tennis players’ career after a doping sanction.

## Methods

The protocol of this observational study was approved by the Ethics Committee of the Nixus Foundation (Permit Number: 2015–08). Informed consent was not required but sanctioned subjects’ were de-identified prior to analysis.

The 2009 World Anti-Doping Agency Code (World Antidoping Agency 2015) defines ‘doping’ as the occurrence of one or more of the anti-doping rule violations, such as: (1) presence of a ‘prohibited substance’, or its metabolites or markers in an athlete’s sample; (2) use or attempted use by an athlete of a ‘prohibited substance’ or a ‘prohibited method’; (3) refusing or failing without compelling justification to submit to sample collection; (4) violation of applicable requirements regarding athlete availability for ‘out-of-competition’ testing; (5) tampering or attempted tampering with any part of doping control; (6) possession of ‘prohibited substances’ and ‘prohibited methods’; (7) trafficking or attempted trafficking in any ‘prohibited substance’ or ‘prohibited method’; (8) administration or attempted administration to any athlete ‘in-competition’ of any ‘prohibited method’ or ‘prohibited substance’, or administration or attempted administration to any athlete ‘out-of-competition’ of any ‘prohibited method’ or any ‘prohibited substance’ that is prohibited ‘out-of-competition’, or assisting, encouraging, aiding, abetting, covering up or any other type of complicity involving an anti-doping rule violation or any attempted anti-doping rule violation.

The International Tennis Federation has published the complete list of anti-doping offences between 2003 and 2014 (International Tennis Federation 2015). According to the doping definition, all offences to the WADA Code committed by tennis players during that period were collected from the ITF official webpage, registered and analyzed. Data of players’ performance (ranking position, retirement date, etc.) were tracked from the Association of Tennis Professional (ATP) official website (Association of Tennis Professionals 2015).

When the original sanction was modified, usually due to a legal appeal by the player, the final decision was considered for analysis.

The career of professional tennis players is measured by the achievement of ranking positions (Reid et al. 2014). Male professional tennis is a highly competitive sport ranked through an objective, merit-based, mathematical system. Since 1973, the Association of Tennis Professionals publishes weekly lists a 52-week rolling computer ranking points based on tournament category. Therefore, the ranking position is a valid instrument to track tennis players’ performance.

Three ranking positions were determined for analysis: (1) the ranking position at the date of doping sanction (DS-P); (2) the highest ranking position after doping sanction (AD-HP); and, (3) the career highest ranking position (C-HP).

The ATP tour published two different rankings for singles and doubles competitions. For the purpose of this study, sanctioned players were defined as “singles player” or “doubles players” according to their best position in both lists. Players’ retirement date were determined through the last appearance in ATP rankings.

Descriptive statistics were obtained and Chi square tests were performed for comparing data from different groups within samples. Wilcoxon paired test was used for comparison of ordinal variables. P values <0.05 were considered significant. (Statistical package: *Statistica for Windows, Statsoft*^*®*^*Tulsa, Oklahoma, USA).*

## Results

Fifty-five doping offences were reported during the 2003–2014 period. Eight cases of doping violations were committed by players without professional tennis records in the ATP ranking. Therefore, 47 cases were included in the final sample of the present study (30 singles players and 16 doubles players). One singles player was charged with two anti-doping rule violations.

The mean age of players at the date of the doping sanction was 26.40 ± 3.48 years (CI 95 %: 25.38–27.42; range 16–34). Only one player (2.12 %) was under 22 years of age at the time of doping sanction.

The average ineligibility time of the doping sanction was 11.13 ± 9.90 months (CI 95 %: 8.23–14.04; range 0–30).

The C-HP of suspended players averaged 201.12 ± 293.96 (n = 47; CI 95 %: 114.81–287.44; range 5–1508; median 83).

The DS-P averaged 409.53 ± 437.38 (n = 47; CI 95 %: 281.11–537.95; range 10–1758; median 266). DS-P data distribution is shown in Table 1. Only 1 player (2.12 %) in the top-10° ranking position was charged with an anti-doping rule violation; elite players committed less doping offences than players beyond the 100° ranking position (29.78 and 70.21 %, respectively; p = 0.0002). If we consider those 8 cases of non-ranked players who were not included in the sample of the present study, the percentage of low-level players would be even greater.

**Table 1:** Frequency distribution of players’ ranking position at the doping sanction date

ATP ranking position	n	Percentage (%)
Top 10°	1	2.12
Position 11°–50°	7	14.89
Position 50°–100°	6	12.76
Position 101°–500°	21	44.68
Position 501°–1000°	7	14.89
Position +1000°	5	10.63
Full ranking	47	100.00

The AD-HP averaged 350.85 ± 441.85 (n = 47; CI 95 %: 221.11–480.58; range 5–1747; median 156). Players were able to improve their ranking position after the sanction (p = 0.0001, Wilcoxon paired test). However, the C-HP was lower than AD-HP (201.12 ± 293.96 vs. 350.85 ± 441.85, respectively; p < 0.0001, Wilcoxon paired test).

Most players (72.34 %) reached their C-HP before the doping sanction, while only 27.65 % got their peak career performance after being suspended (p = 0.01, Chi square test). Double players were more likely to reach their C-HP after doping sanction than singles players (37.5 vs. 22.58 %, respectively; p = 0.27, Chi square test).

The average time to retirement from professional activity of sanctioned players after the doping violation was 35.76 ± 31.03 months (n = 25; CI 95 %: 23.23–48.30; range 0–112). Twenty-two players were still active after the sanction. Twelve percent (6/47) of sanctioned players did not return to professional tennis competition after the doping offence.

Table 2 summarized statistics of the effect of doping sanctions on male professional tennis players.

**Table 2:** Summary of statistics of the effect of doping sanctions on male professional tennis players

	Mean
Average ranking position at sanction date	409°
Average highest career ranking position of sanctioned players	201°
Sanctioned players in the top-10°	2.1 %
Sanctioned players in the top-100°	29.7 %
Direct retirement after doping sanction	12.7 %
Players’ age at sanction date	26.4 years
Average ineligibility time	11.1 months
Players to reach highest career ranking after doping	27.6 %
Time to retirement after doping sanction	35.7 months

## Discussion

Main findings of the present study showed that elite tennis players are less likely to commit a doping offence than low-level players, and that a minority of players suspended from participation in professional tennis due to doping rule violation were able to reach their peak performance after the sanction.

Ranking position analysis of suspended players showed that doping is not a relevant issue among elite tennis athletes. Achieving a top-100° ranking is considered one of the most important markers in professional tennis given the differential benefits that these players obtained in comparison to others who do not get that threshold (Kovacs et al. 2015). Results of the present study showed different indicators revealing that elite tennis players are less likely to commit a doping offence. Ranking position of players at the date of sanction averaged 409° and the highest ranking position of these athletes across their professional career averaged 201°. Most sanctions (70 %) were charged to players beyond the top-100° ranking position and only one player (2 %) was in the top-10° position. It is worth to mention that the majority of anti-doping samples are usually obtained at major tournaments (Maquirriain 2010); therefore, elite tennis players are tested more often that low-level athletes. It is difficult to compare data of doping offences in tennis to other sports; however, since the current anti-doping system was installed in 2003, there was only one doping violation in major tournaments such the Grand Slams and no positive cases in the Olympic Games.

The mean age of players at the sanction date was 26 years and only one player (2.12 %) was under 22 years old. Given that the age of peak performance of tennis players is around 24 years (Maquirriain and Segal 2005), results of this study suggests that tennis players are more likely to commit a doping rule violation in the decline phase of their careers. Athletes are able to carry out proper rational risk assessment about the decision whether or not to take prohibited drugs (Maennig 2014). Athletes’ age may influence that decision and they may be more prone to doping at the decline phase of the sport career (“end-game effect”). Existing punishment mechanisms, such as exclusion from participation in tournaments, have less credible sanctioning effect on an old athlete who is close to his retirement. The athletes` opportunity costs of not being able to earn prize money, his increasing loss of value as he advances in age, may serve as incentives to take banned substances (Dimant and Deutscher 2015).

A substantial percentage of suspended players did not return to professional competition after the sanction. Sekulic (2011) has mentioned that in the case of a positive drug test, the athlete carries a stigma that may lead to a probable career termination. In these terms, for 12.76 % of players in this group, the doping sanction was a true career ending incident.

In summary, the present study analyze professional tennis players’ career after a doping sanction. According to the ranking position at sanction date and their highest career position, most suspended tennis players were not elite players. Doping sanction seems to significantly affect male tennis players’ career. Only a minority of sanctioned players could reach their peak ranking position after the doping offence and suspension may accelerate the retirement process.
